# Entomological profile of yellow fever epidemics in the Central African Republic, 2006–2010

**DOI:** 10.1186/1756-3305-5-175

**Published:** 2012-08-16

**Authors:** Carine Ngoagouni, Basile Kamgang, Alexandre Manirakiza, Auguste Nangouma, Christophe Paupy, Emmanuel Nakoune, Mirdad Kazanji

**Affiliations:** 1 Institut Pasteur de Bangui, PO Box 923, Bangui, Central African Republic; 2 Ministère de la Santé Publique de la Population et de Lutte contre le Sida, PO Box 883, Bangui, Central African Republic; 3 Centre International de Recherches Médicales de Franceville, PO Box 769, Franceville, Gabon

**Keywords:** Yellow fever, Outbreak, Vector, *Aedes*, Central African Republic

## Abstract

**Background:**

The causative agent of yellow fever is an arbovirus of the Flaviviridae family transmitted by infected *Aedes* mosquitoes, particularly in Africa. In the Central African Republic since 2006, cases have been notified in the provinces of Ombella-Mpoko, Ouham-Pende, Basse-Kotto, Haute-Kotto and in Bangui the capital. As the presence of a vector of yellow fever virus (YFV) represents a risk for spread of the disease, we undertook entomological investigations at these sites to identify potential vectors of YFV and their abundance.

**Findings:**

Between 2006 and 2010, 5066 mosquitoes belonging to six genera and 43 species were identified. The 20 species of the *Aedes* genus identified included *Ae. aegypti*, the main vector of YFV in urban settings, and species found in tropical forests, such as *Ae. africanus*, *Ae. simpsoni*, *Ae. luteocephalus*, *Ae. vittatus* and *Ae. opok*. These species were not distributed uniformly in the various sites studied. Thus, the predominant *Aedes* species was *Ae. aegypti* in Bangui (90.7 %) and Basse-Kotto (42.2 %), *Ae. africanus* in Ombella-Mpoko (67.4 %) and Haute-Kotto (77.8 %) and *Ae. vittatus* in Ouham-Pende (62.2 %). *Ae. albopictus* was also found in Bangui. The distribution of these dominant species differed significantly according to study site (*P* < 0.0001). None of the pooled homogenates of *Aedes* mosquitoes analysed by polymerase chain reaction contained the YFV genome.

**Conclusion:**

The results indicate a wide diversity of vector species for YFV in the Central African Republic. The establishment of surveillance and vector control programs should take into account the ecological specificity of each species.

## Findings

### Background

Yellow fever is an acute, often fatal infectious disease caused by a flavivirus (Flaviviridae family) transmitted by mosquitoes and occurring in sub-Saharan Africa and tropical America. Each year, yellow fever virus (YFV) causes an estimated 200 000 cases, of which about 30 000 are fatal, even though an effective vaccine exists. The number of cases has increased over the past two decades [1], perhaps because of decreased population immunity to this infection, deforestation, urbanization, population migration from rural to urban areas and vice versa and the appearance of new vectors. It is therefore crucial to implement vector control operations [2] in order to limit human–vector contact.

In Africa, yellow fever is endemic in 34 countries and continues to cause severe morbidity and mortality [3]. YFV occurs naturally in an enzootic cycle involving monkey populations such as *Cercopithecus aethiopsC. nictitans, Colobus polykomos* and *Papio doguera*[4] and sylvatic mosquito species such as *Aedes africanus, Ae. opok, Ae. simpsoni, Ae. luteocephalus, Ae. taylori* and *Ae. vittatus*, which breed in natural sites (e.g. bamboo stumps, bromeliads and tree holes) [5]. In human settlements, YFV is transmitted during epidemics by *Ae. aegyptiAe. africanusAe. opokAe. simpsoni, Ae. luteocephalus, Ae. metallicus, Ae. furcifer* and *Ae. vittatus* in rural areas and *Ae. aegypti* in urban settings [3]. *Ae. aegypti* is highly anthropophilic and is considered to be the main epidemic YFV vector. Immature stages breed in artificial containers such as used tyres, tin cans and water-storage containers [6,7].

Yellow fever is endemic in the Central African Republic (CAR), where the first case was reported in 1938 [8]. Subsequently, YFV has been isolated repeatedly from human sera [9] and mosquitoes [10] throughout the country. Between 2000 and 2005, only one case of YFV was reported [3]; however, since 2006, several cases have been reported in five regions (Ombella-Mpoko, Ouham-Pende, Basse-Kotto, Haute-Kotto and in Bangui, the capital) on the basis of the presence of IgM antibodies [11]. As the presence of potential vectors of YFV represents a risk for spread of the disease, we undertook entomological investigations at these sites to identify potential vectors of YFV and their abundance.

### Methods

**Figure 1:**
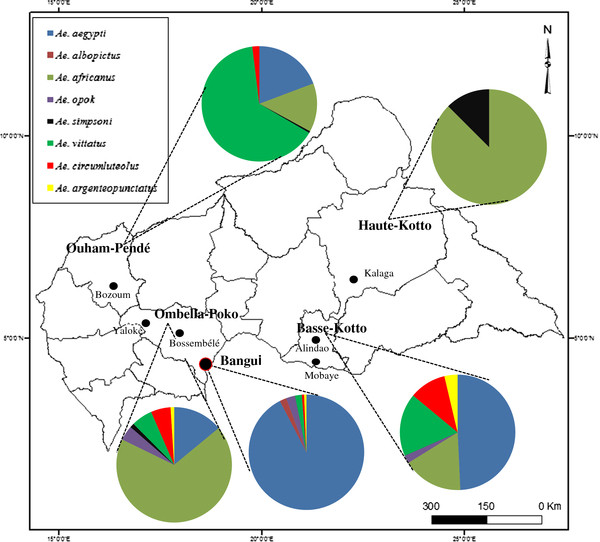
Entomological study sites in Central African Republic and proportions of main vectors of yellow fever virus identified during outbreaks in 2006–2010.

Entomological investigations were conducted between 2006 and 2010 in the five regions of the CAR in which YFV was detected. During the study period, two cases were notified in each of three sites (Bangui, Haute Kotto and Ouham-Pende), four in Basse-Kotto and 32 in Ombella-Mpoko (unpublished data). The geographical locations and main characteristics of the sampling sites are summarized in the Additional file 1 as well as in Figure 1. At each locality, mosquito sampling was started in the households of infected people and then in surrounding households. In Bangui, 100 households around each index case were investigated, while all households were surveyed in the other localities, which were small villages.

In the field, the entomological investigation consisted of a complete inventory of potential larval breeding sites (natural, peri-domestic and domestic) and positive sites (with at least one *Aedes* larva or pupa). Immature stages were collected from positive sites, recorded, transported to the entomology laboratory of the Institut Pasteur in Bangui (IPB) and reared to adult stage for identification. Adult mosquitoes were collected outdoors and in peri-domestic areas during the day after landing on volunteers vaccinated against yellow fever and who were taking malaria prophylaxis. Informed consent was obtained from each volunteer before their inclusion in the study. The investigation of the YFV outbreak was approved by the Ministry of Health and by the national ethical committee of the CAR.

Adult mosquitoes were collected by eight volunteers on 4 days per site between 16:00 and 20:00 h. Mosquitoes collected from humans and those that emerged from immature stages were identified live under a binocular magnifying glass by the morphological criteria described previously [12,13]. After identification, they were anaesthetized by placing them for about 30 min at −20 °C, grouped in single-species pools of up to 10 individuals and stored in a freezer at −20 °C until their arrival at the IPB laboratory, where they were stored at −80 °C.

The YFV genome was sought in all species previously described as YFV vectors by reverse transcriptase polymerase chain reaction (RT-PCR) according to the protocol of Heraud et al. [14].

The numbers of mosquitoes and the frequency of each species were entered on to Excel sheets, and the findings are presented as the proportion of each vector at the localities surveyed. The *χ*^2^ test was used to compare dominant mosquitoes at each site. We hypothesized that the abundance of vectors is a proxy for the number of notified YFV cases per site. Hence, we undertook a trend analysis (*χ*2 test for trends) of the abundance of mosquitoes and the number of cases of YFV notified in Bangui, Ombella-Mpoko, Ouham-Pende and Basse-Kotto. The data for Haute-Kotto were not taken into account in the analysis because only seven specimens of major *Aedes* species were captured.

### Results and discussion

We identified 5066 mosquitoes belonging to six genera and 43 species (Table 1) at the five sites. All the specimens were captured by human landing, except in Bangui (Gobongo 2), where 181 (18 %) specimens in immature stages were found during the survey in five of 16 containers in which water (car wrecks, used tyres, flower pots, tin cans and buckets).

**Table 1:** Culicidae species collected during epidemics of yellow fever in the Central African Republic in 2006–2010

**Species**	**Ombella-Mpoko**	**Ouham-Pende**	**Basse-Kotto**	**Haute-Kotto**	**Bangui**	**Total**
*Aedes aegypti*	142	79	67	0	146	434
*Ae. albopictus*	0	0	0	0	3	3
*Ae. africanus*	691	56	23	7	0	777
*Ae. luteocephalus*	4	0	0	0	0	4
*Ae. domesticus*	2	1	0	0	1	4
*Ae. opok*	42	0	3	0	4	49
*Ae. simpsoni*	12	2	0	1	0	15
*Ae. dentrophilus*	0	1	0	0	0	1
*Ae. vittatus*	60	265	24	0	3	352
*Ae. cumminsi*	0	13	2	0	2	17
*Ae. circumluteolus*	56	8	14	0	1	79
*Ae. haworthi*	0	7	0	0	0	7
*Ae. palpalis*	0	1	0	0	0	1
*Ae. tarsalis*	1	5	0	0	0	6
*Ae. stockesi*	0	1	0	0	0	1
*Ae. argenteopunctatus*	10	0	5	0	1	16
*Ae. subargenteopunctatus*	0	0	2	0	0	2
*Ae. longipalpis*	5	0	0	0	0	5
*Aedes* sp*.*	0	0	5	0	0	5
*Ae. ingrani*	6	0	0	1	0	7
*Culex poicilipes*	23	1	0	0	6	30
*Cx. tigripes*	0	11	0	0	0	11
*Cx. quinquefasciatus*	20	189	54	0	718	981
*Cx. perfuscus*	45	16	17	45	65	188
*Cx. annulioris*	19	0	25	305	0	349
*Culex* sp*.*	109	0	0	0	0	109
*Anopheles paludis*	83	20	2	85	0	190
*An. implexus*	22	0	0	0	0	22
*An. funestus*	43	1	0	5	1	50
*An. gambiae* s.l.	51	4	1	6	13	75
*An. annulioris*	2	0	0	0	0	2
*An. natalensis*	0	0	0	4	5	9
*An. bambusae*	10	0	0	0	0	10
*An. nili*	2	0	0	0	0	2
*An. coustani*	28	16	0	64	2	110
*Mansonia uniformis*	27	80	121	337	4	569
*Ma. africana*	35	44	214	165	10	468
*Eretmapodites chrysogaster*	37	0	18	0	0	55
*Er. inornatus*	24	2	2	0	10	38
*Coquelletidia fraseri*	4	0	0	0	0	4
*Co. cristata*	1	0	1	0	1	3
*Co. pseudoconapas*	4	0	0	0	0	4
*Coquelletidia* sp*.*	0	2	0	0	0	2
**Total**	1620	825	600	1025	996	5066

As seen in Table 1, the species recorded, in order of abundance, were *Culex quinquefasciatus* (19.3 %), followed by *Ae. africanus* (15.3 %), *Mansonia uniformis* (11.2 %), *Ma*. *africana* (9.2 %), *Ae. aegypti* (8.6 %), *Ae. vittatus* (6.9 %), *Cx. annulioris* (6.8 %), *Anopheles paludis* (3.8 %), *Cx. perfuscus* (3.7 %) and others (< 3 %). Three specimens of *Ae. albopictus* were identified only at the two sites in Bangui.

Of the 43 species of mosquito identified, 20 (46.5 %) belonged to the *Aedes* genus. The distribution and abundance of these species varied from one site to another: 11 were found in Ombella-Mpoko and Ouham-Pende, nine in Basse-Kotto, three in Haute-Kotto and eight in Bangui. *Ae. aegypti*, *Ae. africanus*, *Ae. vittatus* and *Ae. circumluteolus* species were present at virtually all the sites investigated, but in different proportions (Figure 1). The predominant *Aedes* species was *Ae. aegypti* in Bangui (90.7 %) and Basse-Kotto (42.2 %), *Ae. africanus* in Ombella-Mpoko (67.4 %) and Haute-Kotto (77.8 %) and *Ae. vittatus* in Ouham-Pende (62.2 %). These dominant *Aedes* species represent 87.7 % of the *Aedes* genus. Their distribution differed widely according to study site (*χ*^2^ = 1166.465, degrees of freedom = 8, *P* < 0.0001).

YFV was not detected by PCR in 184 single-species pools of collected vectors, even though half the *Aedes* species we identified are known to be urban (*Ae. aegypti*), while the others are rural and/or sylvan (e.g. *Ae. africanus, Ae. simpsoniAe. luteocephalus, Ae. vittatus* and *Ae. opok*) YFV vectors [3,5]. The presence of these vectors suggests a high entomological risk for the transmission of YFV in CAR, even though no relation between the abundance of these vectors and the number of reported YFV cases was found (*χ*^2^ test for trend = 1.84, *P* = 0.17). Furthermore, none of the other residents of villages in which cases were found had IgM antibodies against YFV. Therefore, the people who contracted the disease must have been infected elsewhere, probably deep in the forest rather than in their immediate environment. Most of the cases were in hunters, who are in frequent contact with the vectors. Furthermore, the epidemiological investigation showed that the patients started to experience fever in the forest, before they returned to their village health centre.

The disparate distribution of these species in the various sites appears to be due to different environmental conditions or microclimatic variation. In the more rural sites, the predominant species were *Ae. vittatus* (Ouham-Pende) and *Ae. africanus* (Haute-Kotto and Ombella-Mpoko). Although Ombella-Mpoko is in a wooded savannah area and Haute-Kotto is grassland, the sites at which the mosquitoes were caught are surrounded by banana plantations. This biotope could explain the predominance of *Ae. africanus* at both sites, as corroborated by previous studies, in which *Ae. africanus* were found predominantly in vegetation surrounded by raffia or banana plantations [15-17]. Although Ouham-Pende is located in a grassland area, it is mountainous and rocky; the higher prevalence of *Ae. vittatus* in this environment is due to the fact that immature stages of *Ae. vittatus* grow preferentially in rock pools [18].

In the city of Bangui, the most urbanized site investigated, and the semi-urban site of Basse-Kotto, the most prevalent *Aedes* species was *Ae. aegypti*, as found in certain urban areas elsewhere [7,19]. Diallo et al. [20] also found a predominance of *Ae. aegypti* in Bangui. Three *Ae. albopictus* were found in Bangui during the survey, perhaps because of the recent introduction of international trade in used tyres, as observed by Reiter [21].

Our study updates the entomological situation of YFV vectors in the CAR; the last survey was reported by Cordellier and Geoffroy [17]. Herve et al. [22] reported the presence of all these vectors except *Ae. albopictus.* Climatic and environmental modifications might favour the establishment of this species, and a large-scale study is under way to determine its current spread, to compare its adaptation with that of the indigenous species, *Ae. aegypti*, and to evaluate its epidemiological importance.

### Conclusion

Our study of the epidemiological distribution of the vectors of YFV in five geographically distinct regions of the CAR indicates a wide diversity of vector species, suggesting that surveillance and vector control programmes should take into account the ecological specificity of each species.

## Competing interests

The authors have no competing financial interests.

## Authors’ contributions

CN, AN, EN designed the study. CN and AN conducted the field surveys. AM performed the statistical analysis. CN analysed samples in the laboratory under the supervision of EN. CN and BK wrote the manuscript. MK and CP revised the manuscript. All authors read and approved the final manuscript.

## Supplementary Material

Additional file 1 **Geographical locations and main characteristics of sampling sites.** (DOC 26 kb). Click here for file
