# Supplementary material for: Entomological profile of yellow fever epidemics in the Central African Republic, 2006–2010
Source: Parasit Vectors. 2012 Aug 16;5:175. doi: 10.1186/1756-3305-5-175 (PMC3436863; doi:10.1186/1756-3305-5-175)
Supplement: Additional file 1 — Geographical locations and main characteristics of sampling sites. (DOC 26 kb). [file 1756-3305-5-175-S1.doc]

**Supplementary data**:

**Geographical locations and main characteristics of sampling sites**

| Province | Locality | GPS coordinates | Type of environment | Notification date | Entomological investigation date |
| --- | --- | --- | --- | --- | --- |
| Ombella-Mpoko | Bossembélé | 5°16'N 17°39' E | Rural | 14 September 2009 | 30 September 2009 |
|  | Yaloké | 5°16'N 17°07'E | Rural | 14 September 2009 | 30 September 2009 |
|  | Bangui (Gobongo I) | 4°22'N 18°33'E | Urban | 28 November 2009 | 27 February–March 2010 |
|  | Bangui (Gobongo II) | 4°22'N 18°33'E | Urban | 12 July 2009 | 25 August–September 2009 |
| Ouham-Pendé | Bozoum | 6°18'N 16°22'E | Semi-urban | 28 March 2008 | 7–17 May 2008 |
| Basse-Kotto | Alindao | 5°03'N 21°12'E | Semi-urban | 16 January 2009 | 12–22 February 2009 |
|  | Mobaye | 4°19'N 21°11'E | Semi-urban | 05 September 2010 | 7–16 October 2010 |
| Haute-Kotto | Kalaga | 6°49'N 22°09'E | Rural | 10 November 2008 | 26 November–6 December 2008 |
